# Keratin 18-deficiency results in steatohepatitis and liver tumors in old mice: A model of steatohepatitis-associated liver carcinogenesis

**DOI:** 10.18632/oncotarget.12325

**Published:** 2016-09-28

**Authors:** Kira Bettermann, Anita Kuldeep Mehta, Eva M. Hofer, Christina Wohlrab, Nicole Golob-Schwarzl, Vendula Svendova, Michael G. Schimek, Cornelia Stumptner, Andrea Thüringer, Michael R. Speicher, Carolin Lackner, Kurt Zatloukal, Helmut Denk, Johannes Haybaeck

**Affiliations:** ^1^ Institute of Pathology, Medical University of Graz, Graz 8036, Austria; ^2^ Institute for Medical Informatics, Statistics and Documentation, Medical University of Graz, Graz 8036, Austria; ^3^ Institute of Human Genetics, Medical University of Graz, Graz 8010, Austria

**Keywords:** Steatohepatitis, Keratin 18 deficiency, Liver tumors, Mallory-Denk bodies

## Abstract

**Backround:**

Steatohepatitis (SH)-associated liver carcinogenesis is an increasingly important issue in clinical medicine. SH is morphologically characterized by steatosis, hepatocyte injury, ballooning, hepatocytic cytoplasmic inclusions termed Mallory-Denk bodies (MDBs), inflammation and fibrosis.

**Results:**

17-20-months-old *Krt18^−/−^* and *Krt18^+/−^* mice in contrast to wt mice spontaneously developed liver lesions closely resembling the morphological spectrum of human SH as well as liver tumors. The pathologic alterations were more pronounced in *Krt18^−/−^* than in *Krt18^+/−^* mice. The frequency of liver tumors with male predominance was significantly higher in *Krt18^−/−^* compared to age-matched *Krt18^+/−^* and wt mice. Krt18-deficient tumors in contrast to wt animals displayed SH features and often pleomorphic morphology. aCGH analysis of tumors revealed chromosomal aberrations in *Krt18^−/−^* liver tumors, affecting loci of oncogenes and tumor suppressor genes.

**Materials and Methods:**

Livers of 3-, 6-, 12- and 17-20-months-old aged wild type (wt), *Krt18^+/^*^−^ and *Krt18^−/−^* (129P2/OlaHsd background) mice were analyzed by light and immunofluorescence microscopy as well as immunohistochemistry. Liver tumors arising in aged mice were analyzed by array comparative genomic hybridization (aCGH).

**Conclusions:**

Our findings show that K18 deficiency of hepatocytes leads to steatosis, increasing with age, and finally to SH. K18 deficiency and age promote liver tumor development in mice, frequently on the basis of chromosomal instability, resembling human HCC with stemness features.

## INTRODUCTION

Non-alcoholic fatty liver disease (NAFLD) is a growing global health problem affecting one third of the adult population in developed countries [1]. Western lifestyle, e.g., diet rich in saturated fats, central obesity and sedentary behavior, increases the risk of the development of fatty liver with eventual progression to non-alcoholic steatohepatitis (NASH). NASH is characterized by steatosis, inflammation and hepatocyte injury, morphologically expressed by ballooning and Mallory-Denk body (MDB) formation, finally leading to fibrosis and cirrhosis [2]. In this setting, HCC may closely recapitulate the morphologic picture of steatohepatitis (SH) in non-neoplastic liver [3]. Keratin 8 (K8) overexpression and disturbance of hepatocyte keratin homeostasis with increased K8:K18 ratio have been reported as features and may play a role in the pathogenesis of SH [4]. Moreover, NASH also represents an important etiology of hepatocellular carcinoma (HCC), sometimes even in the absence of cirrhosis [3].

The sequence of events leading to NASH and finally to HCC is as yet unclear. Therefore, experimental models, which closely resemble morphologic features of human disease, are of considerable value in unraveling complex pathogenic situations in humans. In this context, data will be presented showing that *Krt18* deficiency together with aging provides a decisive environment for the development of SH and hepatocellular neoplasia in mice [5].

## RESULTS

### Light microscopy and immunohistochemistry of non-neoplastic livers from aged mice of different genotypes

**Figure 1:**
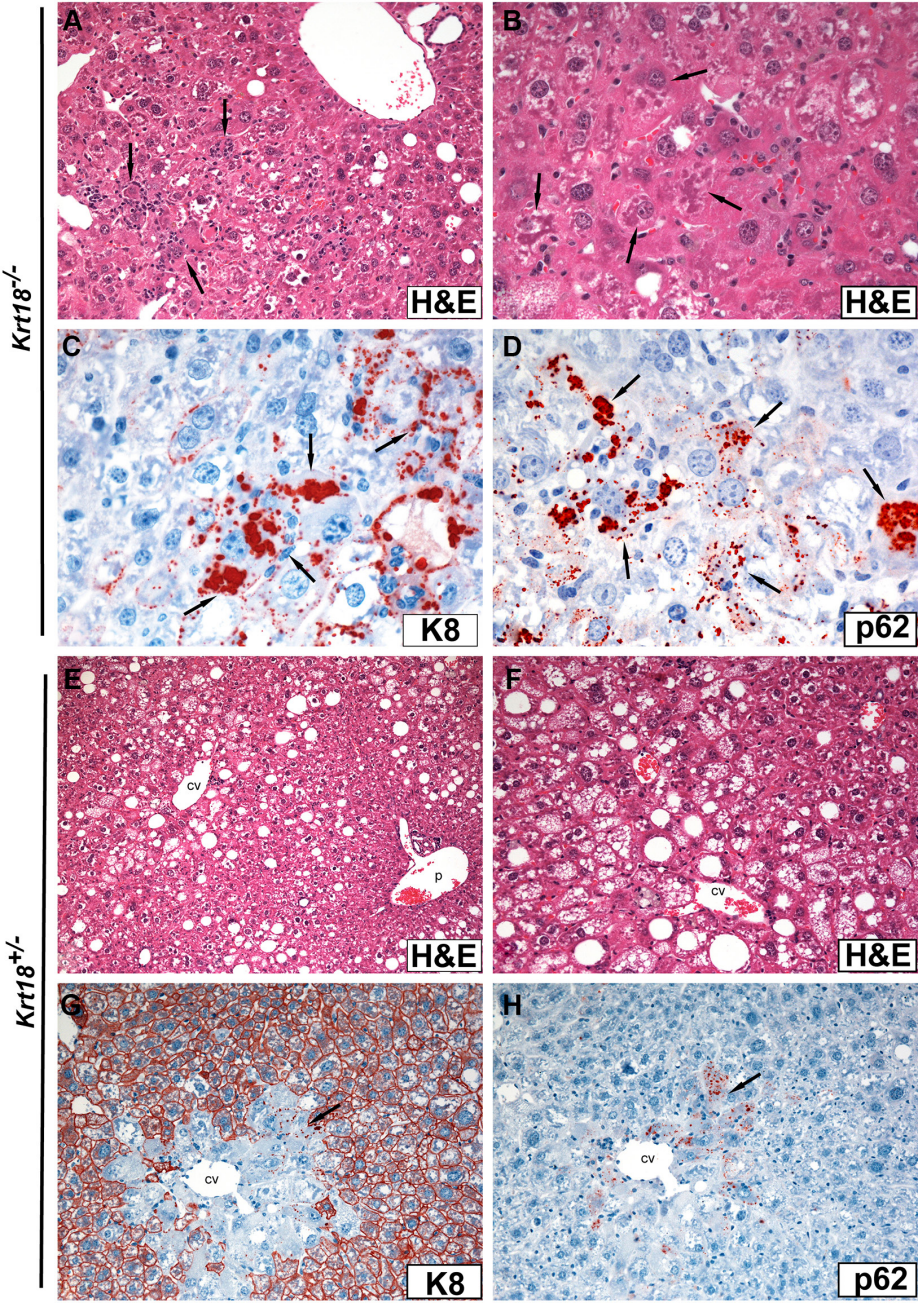
Light microscopy (H&E staining) and immunohistochemistry of livers of aged *Krt18^−/−^* (A–D) and *Krt18^+/−^* (E–H) male mice (**A**) Anisocytosis and anisokaryosis of hepatocytes with cleared-out cytoplasm and mild macrovesicular steatosis. Focal infiltration by mononuclear cells and neutrophils (arrows). (**B**) At higher magnification MDB-containing hepatocytes are indicated by arrows. (**C**) Immunohistochemical demonstration of large and small granular MDBs with antibodies to K8 (arrows). Note that hepatocytes lack K8 immunostaining. (**D**) Immunohistochemical demonstration of large and small granular MDBs with antibodies to p62 (arrows). (**E**) Moderate macro- and microvesicular steatosis predominantly in zones 2 and 3 (cv = central vein; p = portal tract). (**F**) Predominantly microvesicular steatosis (higher magnification; cv = central vein). (**G**) Immunohistochemistry with antibodies to K8: In some centrolobular (perivenular) areas of *Krt18^+/−^* mouse livers the hepatocellular keratin intermediate filament cytoskeleton is greatly reduced or missing (in contrast to the peripheral parenchyma) and small, mostly granular MDBs are decorated with antibodies to K8 (arrow; cv = central vein). (**H**) In a parallel section to (**G**) the granular MDBs are decorated by p62CT antibodies (arrow; cv = central vein).

**Figure 2:**
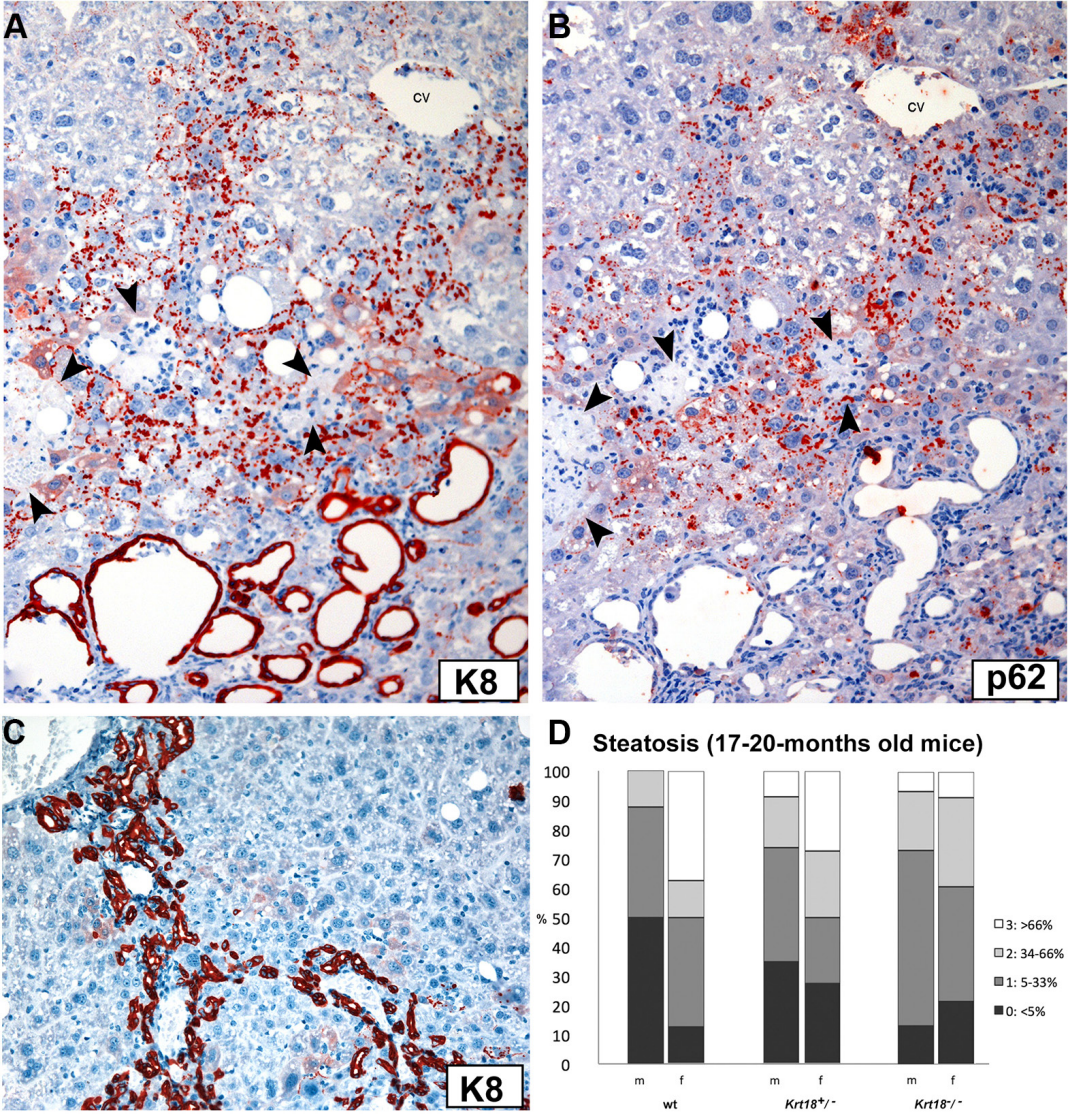
Immunohistochemistry of a parenchymal area in the liver of an old *Krt18^−/−^* mouse with numerous predominantly granular MDBs and focal necrosis of hepatocytes (**A**) Antibodies to K8 decorate granular MDBs (red) and the epithelium of dilated bile ducts (bottom area of the figure). (**B**) The parallel section was immunostained with antibodies to p62CT demonstrating granular MDBs (red). Biliary epithelium is negative (cv = central vein). Necrotic areas in (**A** and **B**) are indicated by arrowheads. (**C**) Pronounced ductular reaction in *Krt18^−/−^* mouse liver as immunohistochemically revealed by K8 antibodies (red). Hepatocytes remain unstained. (**D**) Steatosis grades (percentage) in livers of 17-20-months-old wt, *Krt18^+/−^* and *Krt18^−/−^* mice (m: male, f: female).

Aged male and female *Krt18*^−/−^ mice revealed pronounced hepatocyte anisocytosis and anisokaryosis. Hepatocytes displayed (in H&E stained sections) cleared-out cytoplasm (Figure 1). Focal hemorrhagic parenchymal necrosis, surrounded and penetrated by neutrophils (Figure 2), but also disseminated single hepatocytes containing erythrocytes, as well as syncytial parenchymal areas with nuclear crowding were constant findings. MDBs appeared as irregularly shaped dense eosinophilic cytoplasmic clumps and granules in ballooned hepatocytes, predominantly in perivenular (acinar zone 3) position. By light microscopy combined with immunohistochemistry (see below) they were found in over 90% of the animals irrespective of the gender (Figure 1). Rarely, MDB-containing hepatocytes were surrounded by neutrophils and mononuclear cells (“satellitosis”).

Steatosis was mild to moderate in the majority of the animals irrespective of the gender (Figures 1, 2D), but scores varied considerably in different parts and lobes of the liver. Fatty change prevailed as multiple small and medium-sized cytoplasmic vesicles in zone 3 with gradual coalescence to larger vesicles toward the lobular periphery. Mild lobular inflammatory activity with foci of mononuclear cells was present. Ductular reaction, leading to expansion of portal tracts, and a mild portal mononuclear cell infiltrate surrounding interlobular bile ducts were constant findings (Figure 2C). Thus, the morphology closely resembled SH in humans.

In aged male and female *Krt18^+/−^* mice the degree of steatosis essentially equaled that of *Krt18^−/−^* livers in most animals. However, in some mice (particularly females) it was more conspicuous (Figures 1E, 1F, 2D). Hepatocytes with cleared-out cytoplasm were less abundant and showed zonal (zone 3) and disseminated distribution. In contrast to *Krt18^−/−^* mice, MDB-containing hepatocytes were less frequent: they were found in about 40% of the livers of male *Krt18^+/−^* mice, but only in about 9% of the livers of females. Moreover, MDBs were less well developed, their outline was less distinct, and granular inclusions prevailed, as best revealed by immunohistochemistry (Figure 1G, 1H). With respect to lobular and portal inflammation and ductular reaction no difference existed between homozygous and heterozygous keratin-deficient animals.

In aged wt mice light microscopy disclosed predominantly macrovesicular steatosis in most animals, but with female predominance. SH features were absent. A scarce mononuclear portal infiltrate was almost constantly present.

Apoptotic bodies were rare in livers of aged wt and *Krt18^+/−^*male and female mice, but were slightly more frequent in livers of male *Krt18^−/−^* mice (Supplementary Figure S1).

Immunohistochemistry revealed lack of keratin-specific staining of hepatocytes of *Krt18^−/−^* mice (bile duct epithelia served as positive controls) (Figure 1C, 1D, 1G, 1H), whereas cytoplasmic keratin immunoreactivity with accentuation of the cell periphery was preserved in *Krt18^+/−^* similar to wt animals. MDBs of *Krt18^−/−^* and *Krt18^+/−^* mice were decorated by keratin, ubiquitin (not shown) and p62 antibodies (Figure 1C, 1D, Figure 2) [6, 7]. In *Krt18^+/−^* mice, only in MDB-containing hepatocytes the cytoplasmic keratin immunostaining was diminished or missing (“empty cells”) (Figure 1G, 1H). However, granular inclusions mostly situated at the cell periphery showed less constant immunostaining results: although most showed co-localization of keratin, ubiquitin and p62, some lacked p62 immunoreactivity. On the other hand, strongly p62-positive granular inclusions, often in paranuclear position, lacked keratin- and ubiquitin-related staining and resembled stress-induced p62 aggregates as described previously [8]. Of note, hepatocytes of *Krt18^−/−^* mice showed a slightly increased cytoplasmic background-like staining with keratin antibodies, which was absent in MDB-containing hepatocytes.

In order to monitor the time course of the development of liver lesions with advancing age, 3-, 6-, and 12-months-old wt, *Krt18^+/−^* and *Krt18^−/−^* mice, were included in our study. In 3-months-old wt mice no pathologic alterations were found in the liver except mild (mostly macrovesicular) steatosis in some male animals. In *Krt18^+/−^* animals (particularly in males) hepatocytes, predominantly located in zone 3, showed clear cytoplasm with more intense staining of the cell periphery and mild macrovesicular steatosis. Liver morphology in *Krt18^−/−^*mice was almost identical, but hepatocytes with clear cytoplasm were more frequent and diffusely distributed. Some hepatocytes of *Krt18^−/−^* livers contained erythrocytes in their cytoplasm, and focally syncytial parenchymal areas with crowded nuclei were present [9, 10]. Inflammation, hepatocyte ballooning, MDBs and tumors were absent. No gender difference was recognizable. Identical morphology existed in 6- and 12-months-old animals of the different genotypes. However, the number of animals with fatty liver as well as the steatosis grades increased with age and keratin deficiency status: in 17-20-months-old mice only mild steatosis was found in about one third of wt mice whereas it was mild to moderate in almost all *Krt18^+/−^* and *Krt18^−/−^* mice (Figure 2D). No liver fibrosis occurred.

### Altered liver enzyme levels

Serum aspartate aminotransferase (AST), alanine aminotransferase (ALT), alkaline phosphatase (AP), urea (U), triglyceride (TG) and cholesterol (Chol) levels showed no significant differences between aged wt, *Krt18^+/−^* and *Krt18^−/−^* mice. Still, overall transaminase levels were more elevated in *Krt18^+/−^* and *Krt18^−/−^* as a sign of liver injury (Supplementary Figure S2, Supplementary Table S1).

### Gross pathology, light microscopy and immunohistochemistry of tumors

**Figure 3:**
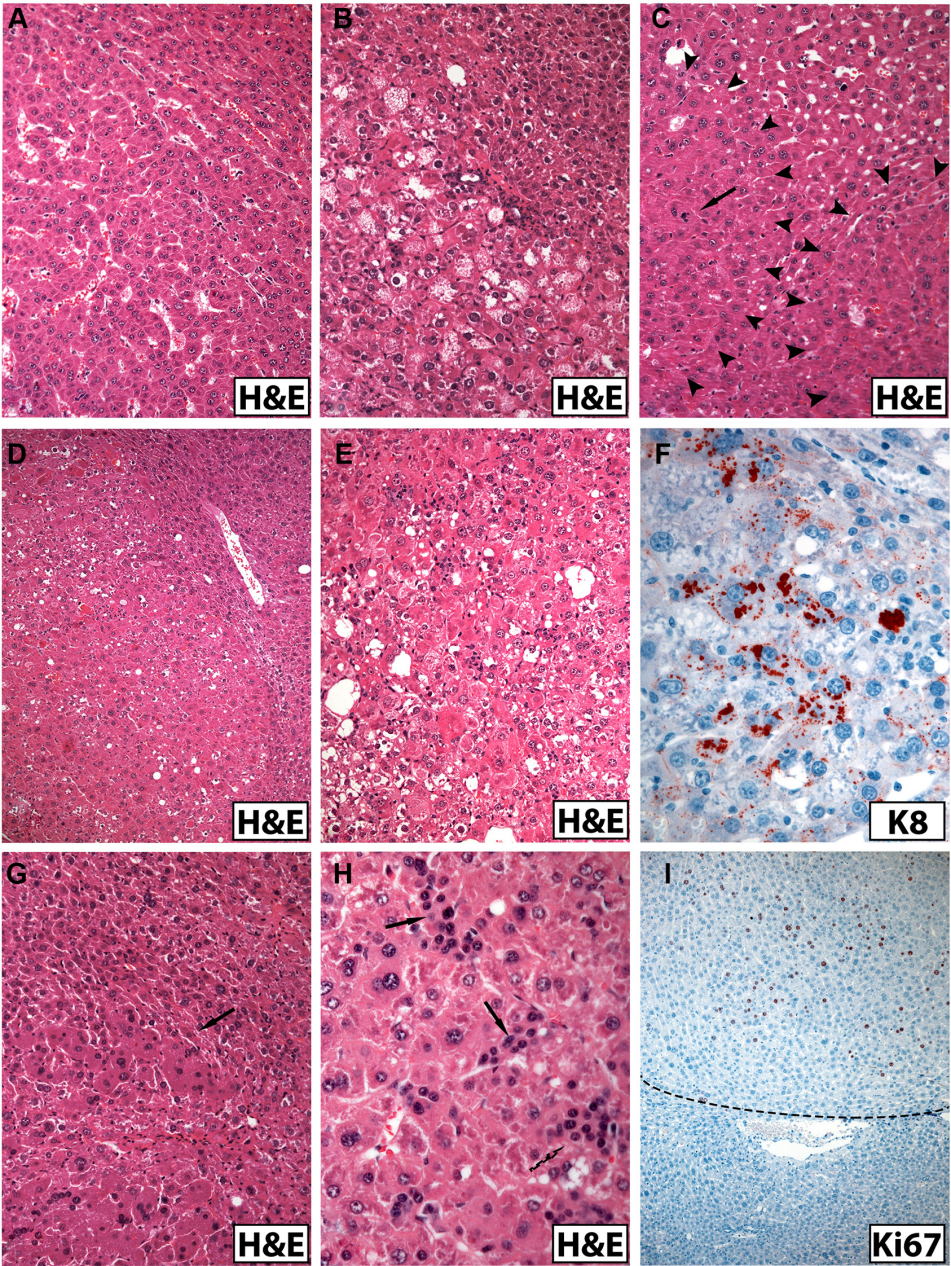
Morphologic spectrum of hepatocellular tumors arising in old male mice (A–E, G, H, H&E staining; immunohistochemistry in F and I) (**A**) Monomorphic trabecular non-encapsulated liver tumor in a male *Krt18^+/−^* mouse. (**B**) Liver tumor in a *Krt18^−/−^* mouse showing a more irregular cell arrangement and microvesicular steatosis. (**C**) Liver tumor in a *Krt18^+/−^* mouse with nodule-in-nodule formation delineated by arrowheads and mitosis (arrow). (**D**) Pleomorphic liver tumor in a *Krt18^−/−^* mouse showing morphologic signs of steatohepatitis. (**E**) Liver tumor from a *Krt18^−/−^* mouse showing cellular atypia and signs of steatohepatitis with MDBs [not easily visible in the H&E stained section, compare with (**F**) and focal inflammatory infiltrates]. (**F**) Same tumor as in (**E**): The presence of larger and granular MDBs is confirmed by immunohistochemistry using K8 antibody (red). (**G**) Areas of large tumor cells with homogeneous oxyphilic cytoplasm in a liver tumor of a *Krt18^−/−^* mouse (arrow). (**H**) Small tumor cells morphologically resembling ductular epithelium (arrows) in a pleomorphic liver tumor of a *Krt18^−/−^* mouse. (**I**) Immunohistochemistry: Increased number of Ki67-positive tumor cell nuclei (*Krt18^+/−^* mouse). The border to the non-neoplastic liver (lower third) is indicated by the broken line.

**Figure 4:**
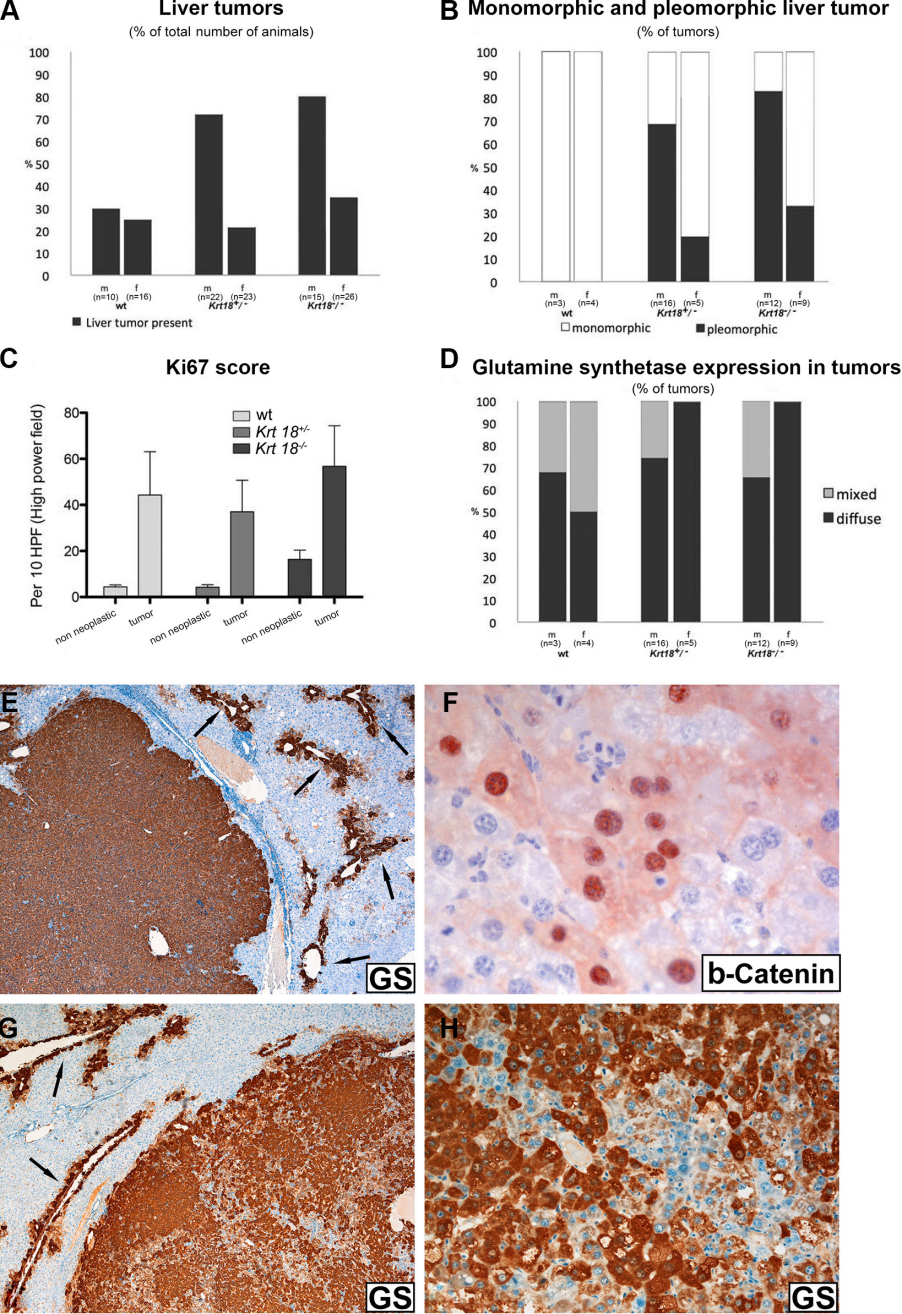
Histopathological characterization of liver tumors of 17-20-months-old wt, *Krt18^+/−^* and *Krt18^−/−^*mice Immunohistochemical demonstration of glutamine synthetase in liver tumors (male 17-20-months-old *Krt18*^−/−^ mice) (**E**–**H**). (**A**) Presence of liver tumors in percent. (**B**) Monomorphic and pleomorphic tumors in percent. (**C**) Ki67 immunoreactivity in tumor and non-neoplastic region. (**D**) Glutamine synthetase positivity of liver tumors in percent (m: male, f: female). (**E**) Homogeneous GS positivity of the well demarcated tumor. The surrounding non-neoplastic liver expresses GS only in a perivenular layer of hepatocytes (arrows). (**F**) Antibodies to beta-catenin reveal immunostaining of the nuclei as well as a faint cytoplasmic decoration of a group of tumor cells. (**G**) Tumor with heterogeneous GS content (arrows indicate GS in surrounding non-neoplastic liver). (**H**) Higher magnification of (**C**) showing GS-positive and negative tumor areas.

Macroscopic and microscopic analysis of aged male and female wt, *Krt18^+/−^* and *Krt18^−/−^* mice revealed liver tumors in animals of every genotype but with different frequencies with clear preponderance of the male gender. In the wt group, tumors were found in ~30% of male and ~25% of female mice, whereas ~73% of male and ~22% of female *Krt18^+/−^* mice and ~80% of male and ~35% of female *Krt18^−/−^* mice harbored liver tumors (Figures 3, 4A). In *Krt18^−/−^* mice we often found multiple tumors. Male *Krt18^−/−^* mice developed significantly more tumors than male and female wt mice (Supplementary Table S2). Tumor sizes of 17-20-months-old wt, *Krt18^+/−^* and *Krt18^−/−^* mice did not significantly differ between the genotypes (Supplementary Figure S3, Supplementary Table S3). Histology confirmed the presence of distinct non-encapsulated nodules which stood out by their expanding growth, leading to compression of the surrounding parenchyma, and irregular arrangement and morphology of tumor cells. Definite signs of invasion were absent. The majority of tumor cells was larger than non-neoplastic hepatocytes, but tumor cells equal in size to or smaller than non-neoplastic hepatocytes were also visible. According to the degree of cellular atypia predominantly monomorphic and pleomorphic tumors were distinguished (Figures 3, 4B). Tumors of wt mice were exclusively monomorphic. The *pleomorphic* type was more frequent in *Krt18^−/−^* mice and clearly prevailed in male (~83% in *Krt18^−/−^* and ~69% in *Krt18^+/−^* animals) as compared to female *Krt18^−/−^* and *Krt18^+/−^* mice (~33% in *Krt18^−/−^* and 20% in *Krt18^+/−^* animals) (Figures 3, 4B).

In *monomorphic* tumors, the tumor cells were more regularly arranged in a trabecular fashion. The cytoplasm of the majority of tumor cells was less eosinophilic in H&E stained sections than non-neoplastic hepatocytes, but also tumor cells with oncocytic appearance were observed. The degree of steatosis varied, but was usually less than in the non-neoplastic parenchyma. The number of apoptotic bodies was increased in all tumors. Morphologic features of SH with MDBs were exclusively present in *Krt18^−/−^* and *Krt18^+/−^* mice (Figure 3D–3F).

In *pleomorphic* tumors, cellular and nuclear pleomorphism was conspicuous. Rarely, cells with bizarre irregular nuclei and atypical mitotic figures were seen. The architecture was more complex, often leading to nodule-in-nodule formation. The latter prevailed in male *Krt18^−/−^* mice, but was also seen in *Krt18^+/−^* and wt animals (Figure 3C). Areas consisting of small tumor cells mimicking ductular epithelium with hyperchromatic nuclei, high nuclear/cytoplasmic ratio and focal solid arrangement with nuclear crowding were also present in most tumors associated with *Krt18*-deficient mice (Figure 3). The number of MDB-containing tumor cells, usually focally concentrated, was increased in *Krt18^+/−^* and *Krt18^−/−^* mice.

The number of Ki67-immunoreactive tumor cell nuclei, indicating mitotic cells, was considerably increased in a focally accentuated fashion in all tumors irrespective of the genotype whereas mitotic activity was low in the non-neoplastic liver parenchyma (Figure 3I), although in areas with morphologic features of SH Ki67-positive hepatocytes were more frequent than in those with less conspicuous alterations (Figure 4C).

Glutamine synthetase (GS) was immunohistochemically detectable in all tumors arising in K18-deficient and in almost all (> 90%) arising in wt livers as intense cytoplasmic staining, in either diffuse or patchy/mixed pattern (Figure 4D–4H) suggesting mutations in the Wnt/beta-catenin signaling pathway [11]. In support of this assumption, immunohistochemical demonstration of beta-catenin revealed nuclear in addition to cytoplasmic staining (Figure 4F). In the surrounding non-neoplastic liver only the perivenular hepatocyte layer was GS-positive, thus serving as positive control (Figure 4E, 4G) [12].

**Figure 5:**
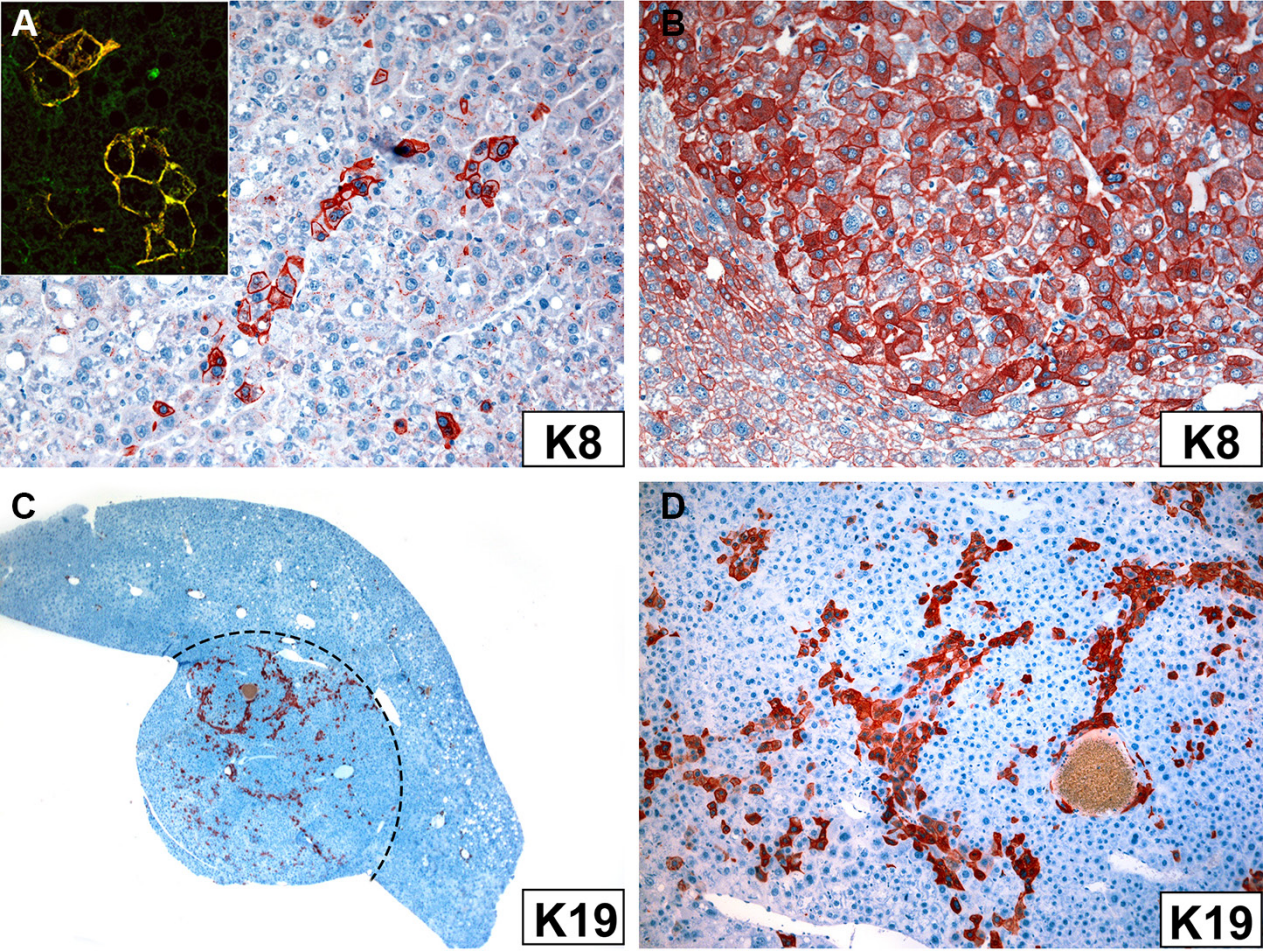
Immunoreactivity of tumor cells in *Krt18^−/−^* (A) and *Krt18^+/−^* mice (B–D) with antibodies to K8 (A, B) and K19 (C, D) (**A**) Small group of K8-positive hepatocytes. Inset: Double-immunofluorescence with antibodies to K8 (green) and K19 (red) shows co-localization indicated by the yellow color. (**B**) Considerable variation in K8 immunostaining intensity of tumor cells. The non-neoplastic cells at the periphery are less intensely stained (lower left corner). (**C**) K19 expressed in tumor cells (red; border of the tumor indicated by broken line). (**D**) Higher magnification of (**C**) showing K19-positive tumor cells mostly arranged in strands.

In immunohistochemistry, the majority of *Krt18^−/−^* tumor cells were, as expected, keratin-negative whereas *Krt18^+/−^* tumor cells were keratin-positive with considerable variation in staining intensity suggestive of variations in IF content and density (Figure 3F, 5A, 5B). It is noteworthy, however, that in some *Krt18^−/−^* liver tumors K8 immunoreactivity was noted in disseminated polygonal tumor cells or small cell groups (5–20 cells), sometimes in trabecular arrangement or in strands, whereas the small cells were negative (Figure 5A). The persistence of K8 in filamentous form could result from the presence of a surrogate keratin I partner such as K19. Consequently, tumors were immunostained with antibodies to K19. Indeed, K19 was co-expressed in these K8-positive tumor cells, as best revealed by double-immunofluorescence microscopy (inset in Figure 5A). K19-positive tumor cells were also present in the majority of tumors arising in *Krt18^+/−^* and wt mouse livers (Figure 5C, 5D). Thus, no correlation existed between the presence of K19-positive cells and the genotype of the animals. If multiple tumors were present in the liver, the individual tumors showed considerable variation in the content of K19-positive cells, and nodules without or with only very few K19-positive cells coexisted with those rich in K19-positive cells. In the presence of MDBs cytoplasmic keratin immunoreactivity was reduced or even abolished as described previously (Figure 1G, 1H) [13]. Classical MDBs and granular aggregates in tumor cells resembled in their immunoreactivity the non-neoplastic situation.

### Genetic liver tumor profiles

**Figure 6:**
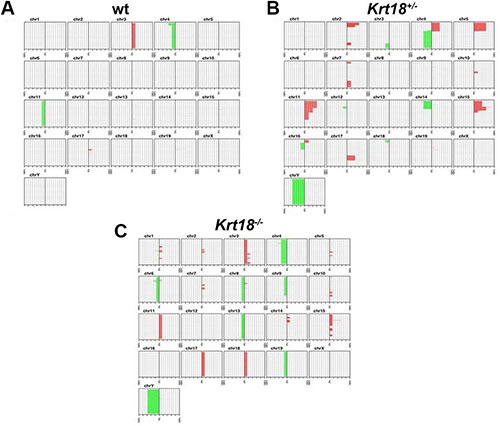
Chromosomal aberration profile of liver tumors of 17-20-months-old wt, *Krt18*^+/−^ and *Krt18*^−/−^ mice Accumulative penetrance plot of chromosomal imbalances in wt, *Krt18^+/−^* and *Krt18^−/−^* mice. Each sub-graph shows the q-arm of one chromosome specified by a number. The horizontal axis in each sub-graph indicates the number of samples, while the vertical axis shows the position on the q-arm for each chromosome. Chromosomal amplifications are indicated in red on the right part of each sub-graph and deletions are indicated in green on the left part of each sub-graph. (**A**–**C**) aCGH reveals more chromosomal aberrations in all *Krt18^−/−^* (**C**) liver tumors than in wt (**A**) and *Krt18^+/−^* (**B**) liver tumors. wt mice: 11 males, 5 females; *Krt18^+/−^* mice: 5 males, 0 female; *Krt18^−/−^* mice: 7 males, 1 female.

**Figure 7:**
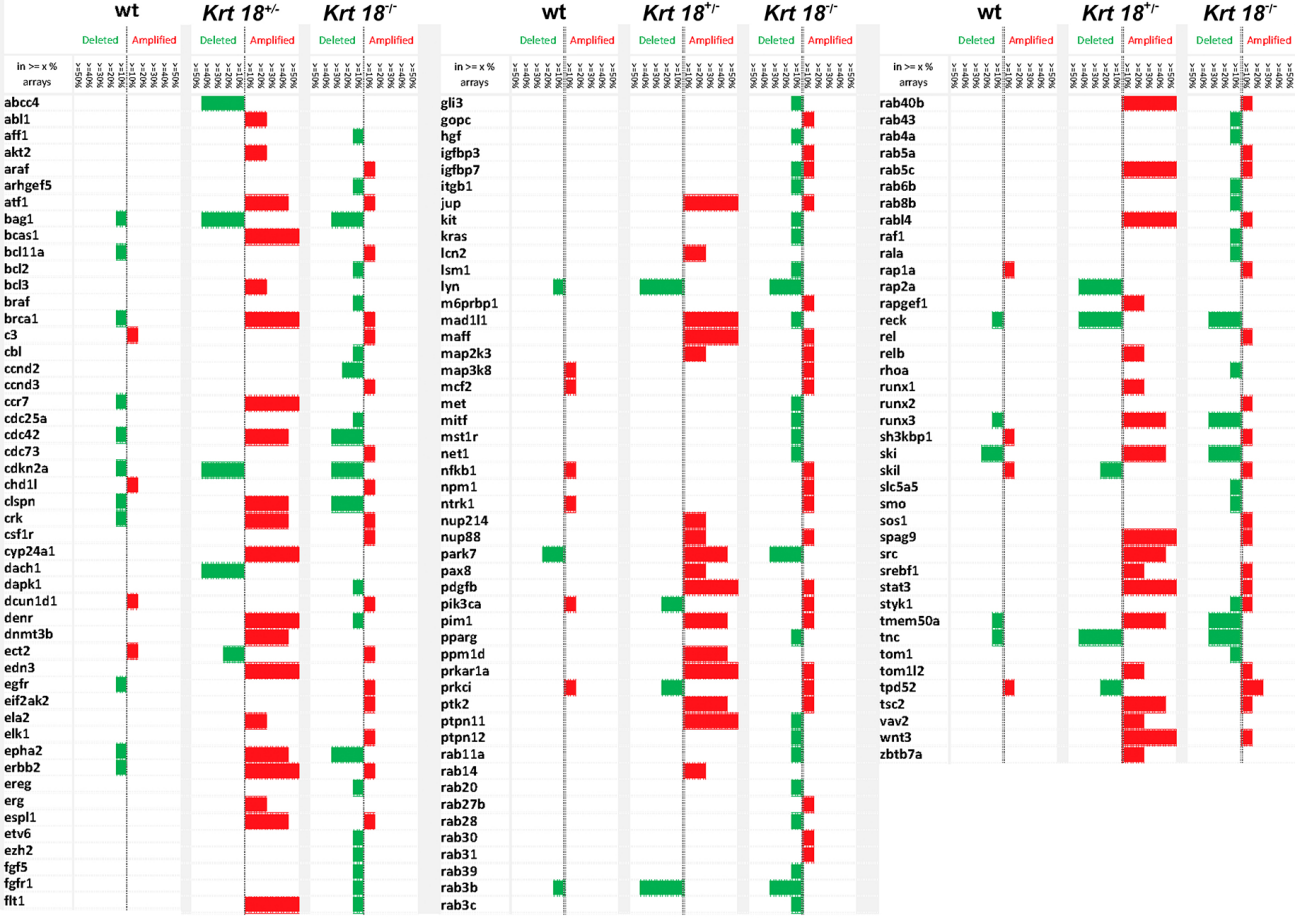
Genetic profile of liver tumors Comparison of the genetic aberrations in wt, *Krt18^+/−^* and *Krt18^−/−^* mice. The height of each bar denotes the percentage of arrays, in which the deletion/amplification occurred (ranging between ‘at least 10%’ and ‘50% or more’ of arrays). wt mice: 11 males, 5 females; *Krt18^+/−^* mice: 5 males, 0 female; *Krt18^−/−^* mice: 7 males, 1 female.

aCGH revealed chromosomal aberrations in all tested tumors of male mice irrespective of the genotype. Amplifications and deletions of chromosomal regions ranged from ≤ 1 megabase (MB) to 160 MB and were detected in most autosomes. However, the highest number of chromosomal deletions and amplifications was found in *Krt18^−/−^* compared to *Krt18^+/−^* and wt tumors. The altered chromosomes contained loci with known oncogenes, tumor suppressor and liver cancer-associated genes (Figures 6 and 7, online Supplementary material and the NCBI Gene Expression Omnibus accession number for the murine HCC data reported is GSE47212).

Based on these data we generated a genetic profile of the tumors by aCGH. This analysis revealed up-regulation of several oncogenes and down-regulation of tumor suppressor genes. Many known human HCC-associated oncogenes were upregulated in *Krt18^−/−^* liver tumors [*bcl11a, ccnd3* [14]*, igfbp3* [15]*, npm* [16]*, rab27b* [17]*, srebf1* [18]*, stat3* [19]*, wnt3* [20]] and known human HCC tumor suppressor genes were down-regulated [*rab11a* [17]*, runx3* [21]] (Figure 7). These changes were not observed in wt tumors.

Chromosomal instability in human cancer is believed to often take place before neoplasms display morphological signs of invasion. In many tumor entities, the number of chromosomal aberrations increases side by side with increasing histological grade and risk of metastasis, reflecting a stepwise accumulation of cytogenetic alterations during tumor progression [22]. To assess the impact of chromosomal instability during aging in liver tumorigenesis we investigated non-tumorous liver tissue of the three genotypes at the age of 12 and 17–20 months by performing additional aCGH analyses. Indeed in some cases of *Krt18^+/−^* and *Krt18^−/−^* livers amplifications and deletions were observed in non-neoplastically transformed liver tissue also at 12 months of age, i.e., before the onset of tumor occurrence but not in wt mice (Supplementary Figure S4, Supplementary Table S4).

## DISCUSSION

We show that aged (17-20-months-old) *Krt18^−/−^* mice spontaneously develop morphologic features of SH as well as liver tumors preceded in younger animals by steatosis as possible “first hit”. This animal model is thus relevant for human non-alcoholic fatty liver disease regarding liver morphology and its impact on carcinogenesis. Like in the human situation, gender disparity exists with male preponderance of liver tumor development, which might be related to the hormonal (estrogen) status of females [23, 24].

It is obvious from our results that two major pathogenic principles act in synergy, namely the disturbance of hepatocellular keratin homeostasis (i.e., the disturbance of the molar ratios of type I keratins, i.e. K18, and type II keratins, i.e. K8) and aging with its metabolic consequences [5, 25]. Aging is characterized by irreversible decline of various physiological functions [5], and impaired resistance to multiple forms of stress [26]. Our results are consistent with the clinical observation that older patients are at increased risk of NAFLD/NASH as well as HCC [3]. Oxidative stress is considered to be a prime player in this context [5, 13].

According to findings in humans and experimental animals, keratins behave in addition to their role as structural proteins to some extent like stress proteins. Their expression levels and posttranslational modifications, particularly hyper-phosphorylation, are affected by various stress conditions. *Krt* genes are regarded as susceptibility genes for a variety of human and experimental liver diseases [27, 28]. *Krt* mutations or imbalanced expression of keratin pairs (i.e., K8 and K18 in hepatocytes) might sensitize the liver to attacks by toxins or viral infections, particularly mediated by oxidative injury [13, 29]. K8 and K18 proteins protect hepatocytes in a non-mechanical way by serving as targets of abnormally activated (stress) kinases and acting as “phosphate sponge/sink” [30]. By that keratins modulate cell signaling pathways, including those involved in cell death, glucose metabolism and protein synthesis, target cellular proteins and protect mitochondria [27, 28]. Furthermore, K18 represents a major caspase substrate during apoptosis and keratins are suggested to modulate apoptosis. Therefore, functional relationships may exist between increased apoptosis in SH and alteration of K18 expression [31].

The liver lesions associated with K18 deficiency provide, on the one hand, evidence for impairment of cellular stability, finally leading to haemorrhagic necrosis of hepatocytes, and, on the other hand, for defective non-mechanical keratin functions. Increased sensitivity to oxidative stress [25, 32] seems to be the prime driving force behind the development of SH with MDB formation. The study by Kucukoglu et al. clearly underlines that excess of K8 primes mice towards SH-like injury [33].

The aggregation of ubiquitinated and hyperphosphorylated keratins together with the stress- and adapter protein p62 and other stress proteins in the form of MDBs could actually represent the phenotypic equivalent of a rescue reaction of the senescent hepatocyte leading to biologically inert inclusions [34]. In several NAFLD models hepatocyte senescence is associated with impaired regeneration and increased risk of neoplasia [35]. Our results expand these observations by showing that disturbance of keratin homeostasis and resulting SH in the aged animal predispose to tumor development. In humans, HCC encompasses a heterogeneous group of subtypes with different morphology, etiologies, environmental risk factors and transformation pathways that are reflected in variable clinical courses [36, 37]. Comparison of the gene signatures of liver tumors associated with wt, *Krt18^+/−^* and *Krt18^−/−^* mice revealed clear-cut differences between wt and *Krt18^−/−^* tumors, whereas tumors arising in *Krt18^+/−^* animals occupied an intermediate position. The tumors found in the old *Krt18^−/−^* mice closely resembled human HCCs in their genetic profile regarding amplifications of chromosomal regions with several oncogenes, proto-oncogenes and loss of tumor suppressor genes [38, 39](Figures 6 and 7). These tumors also morphologically mimicked the steatohepatitic hepatocellular carcinoma (SH-HCC) found in human livers [40].

Cellular atypia, increased mitotic rates and architectural abnormalities, particularly nodule-in-nodule formation, as well as the genetic data suggest malignancy or at least malignant potential particularly of the tumors arising in *Krt18^−/−^* mice. This is in line with the expression of GS, which, together with abnormal beta-catenin immunostaining, suggests activation of the Wnt/beta-catenin pathway. Tumor cells with up-regulation of GS expression appear to possess a certain growth advantage as a consequence of their independence of the supply of glutamine and carry a higher risk of malignant transformation and aggressive clinical behavior [41].

It is further interesting in this context that the majority of tumors associated with aged animals expressed K19 in tumor cells in a disseminated or patchy distribution with considerable variation but without correlation with cellular atypia. In these tumor cells the type I K19 acted as polymerization partner of K8 and prevented its degradation. Moreover, by expressing K19 these tumor cells show features of intermediate cells with a hepatocyte-bile duct cell phenotype. K19 is a biliary/hepatic progenitor cell marker, which is expressed in a subset of human HCCs with poor prognosis [42]. It has been shown that K19 expression in a fraction of tumor cells (> 5%) in surgical specimens of HCC correlated with increased tumor size, decreased differentiation, early metastatic spread and microvascular invasion [42].

The observation that tumors of heterozygous and homozygous *Krt18*-deficient mice showed chromosomal instability, which was not observed in wt mice, suggests a novel mechanism by which keratins might contribute to tumor development. Since some *Krt18*-deficient mice showed chromosomal instability already at the age of 12 months in the tumor-free liver, keratin-related chromosomal instability seems to precede tumor development, and therefore might be a causal factor rather than a secondary event. Furthermore, the fact that both heterozygous and homozygous *Krt18*-deficient mice demonstrate chromosomal instability and increased tumor incidence suggests that it is not caused by the absence of the Keratin IF cytoskeleton (which is only the case in homozygous *Krt18*-deficient mice) but rather the imbalance of Type I and Type II keratin expression. To the best of our knowledge, this is the first time that the imbalance of K8 and 18 or any other keratin pair is shown to have an impact on chromosomal instability. The mechanisms by which keratin imbalance might cause chromosomal instability needs to be elucidated. One possible explanation could be that keratins influence the karyoskeleton. A study by Tolstong et al. [43] points to an interconnection of cytoplasmic IFs and structural elements of the nuclear matrix, and make them, together with their susceptibility to cross-linkage to nuclear matrix attachment regions and other genomic DNA sequences, complementary or integral components of the karyoskeleton. Thus, it was concluded that keratins might preferentially be associated with chromatin within the nucleus [44].

Loss of other IFs, namely A-type lamins has been described to change the nuclear distribution of telomeres resulting in telomere shortening, defects in telomeric heterochromatin, and increased genomic instability [45]. Alterations in the expression of A-type lamins are associated with different types of human tumors, and genomic and chromosomal instability are known to significantly contribute to tumorigenesis and have also been linked to premature aging [46, 47].

Previous studies revealed increased chromosomal instability in human HCCs with “stemness”-related protein expression [46]. Human HCCs expressing K19 or EpCAM demonstrated significantly higher copy number aberrations compared to HCCs not expressing these markers, suggesting increased chromosomal instability in “stemness”-related marker expressing HCCs [47].

In conclusion, disturbance of keratin homeostasis with an excess of K8 causes SH in aged mice and primes for liver carcinogenesis. Interestingly, male gender predisposes to liver tumor formation in this animal model, as also observed in human patients. The observations made in aged *Krt18^−/−^* mice suggest that this is a novel model sharing several key features of human disease for further dissecting molecular pathways between SH and HCC.

## MATERIALS AND METHODS

### Experimental animals

Our studies focused on aged (17- to 20-months-old) wild type (wt), heterozygous (*Krt18^+/−^*) and homozygous (*Krt18^−/−^*) K18-deficient mice [48, 49]; 3-, 6- and 12-months-old animals were included for comparison and to monitor the time course of the pathologic process. Mice were maintained under specific pathogen-free (SPF) conditions in a temperature-controlled environment (20–24°C, humidity 50–60%) with 12 hours light-dark rhythm; they received a standard diet (ssniff Spezialdiäten GmbH, Soest, Germany) and water ad libitum. Serum enzymes and other serum components as indicators of liver cell damage were determined by routine procedures. Experimental protocols were in accordance with the Austrian Animal Protection Law, Veterinary office, Vienna. The study was approved by the institutional ethics committee and animal experiment license granted under No. BMWF-535233.

### Measurement of mouse serum aminotransferases

Serum levels of AST, ALT, AP, Chol, HST and TG were measured using enzymatic reagents (Roche Diagnostics, Mannheim, Germany) on a cobas analyzer (Roche Diagnostics).

### Light microscopy

Mice were sacrificed by cervical dislocation, liver tissue samples were fixed in 4% buffered formaldehyde solution, embedded in paraffin; dewaxed 7 μm thick sections were stained with hematoxylin and eosin (H&E). Apoptotic hepatocytes were identified by their morphological features, i.e., eosinophilic cytoplasm and shrunken, fragmented or absent nuclei and counted per high-power fields (HPF; 400× magnification) in different areas of the liver section to account for heterogeneous distribution. In every liver 10 HPFs were evaluated.

### Immunohistochemistry

Antibodies and immunostaining conditions are listed in Supplementary Table S5. 3 μm thick dewaxed sections of formaldehyde-fixed and paraffin-embedded liver tissue were treated as described previously [48, 50].

### Immunofluorescence microscopy

Frozen tissue sections were fixed in −20°C cold acetone for 10 minutes and dried. Samples were processed as described [8]. Primary antibody CK8 Troma (Progen) was applied for 30 minutes, slides were washed with PBS buffer, followed by Alexa Fluor 488 goat anti-rat IgG (H+L; 1:500) (Invitrogen A11006, Carlsbad, CA, USA) for 30 minutes in the dark. Subsequently PBS was applied, then aqua dest. and briefly 100% alcohol, followed by drying and mounting with Moviol. p62 was detected by using a rabbit antibody to the C-terminus of p62 [51] and Rhodamine Red-X-conjugated goat anti-guinea pig immunoglobulin (1:5000 Jackson Immune Research, West Grove, PA, USA).

### Array comparative genome hybridization (aCGH)-analysis and respective statistical analysis

Tumor tissue was isolated from the paraffin blocks by micro-dissection and genomic DNA was prepared [52]. It was hybridized against DNA of age-matched non-neoplastic wt livers of male mice and further processed according to the Agilent protocol (Version 7.2, Agilent, Santa Clara, CA, USA). The data were analyzed using the statistical software R, applying MSMAD [53].

### Statistical analysis for evaluation of Ki67 and apoptotic bodies

A chi-square test with a significance level of *p* = 0.05 was used. Data are reported as median values with 95% confidence interval limits. They were assessed by non-parametric bootstrap using DATAPLOT (National Institute of Standards and Technology, Statistical Engineering Division, Gaithersburg, MD, USA) and GraphPadPrism software. A two-way-ANOVA with Bonferroni post-testing performed to evaluate the significance of differences between groups.

### Statistical analysis for evaluation of tumor counts and tumor sizes

The tumor counts (Supplementary Table S2) and tumor sizes (Supplementary Table S3) were evaluated using Wilcoxon rank sum test. The significance level was set to *p* = 0.05.

### External data bases

The NCBI Gene Expression Omnibus accession number for the murine HCC development: comparison of wt, *Krt18^+/−^*, *Krt18^−/−^* liver tumors hybridized against non-affected wt liver tissue is GSE81054.

## SUPPLEMENTARY MATERIALS TABLES AND FIGURES


