# Supplementary material for: Keratin 18-deficiency results in steatohepatitis and liver tumors in old mice: A model of steatohepatitis-associated liver carcinogenesis
Source: Oncotarget. 2016 Sep 28;7(45):73309–22. doi: 10.18632/oncotarget.12325 (PMC5341981; doi:10.18632/oncotarget.12325)
Supplement: Supplementary file 1 [file oncotarget-07-73309-s001.pdf]

Keratin 18-deficiency results in steatohepatitis and liver tumors in old mice: A model of steatohepatitis-associated liver carcinogenesis

Supplementary Materials

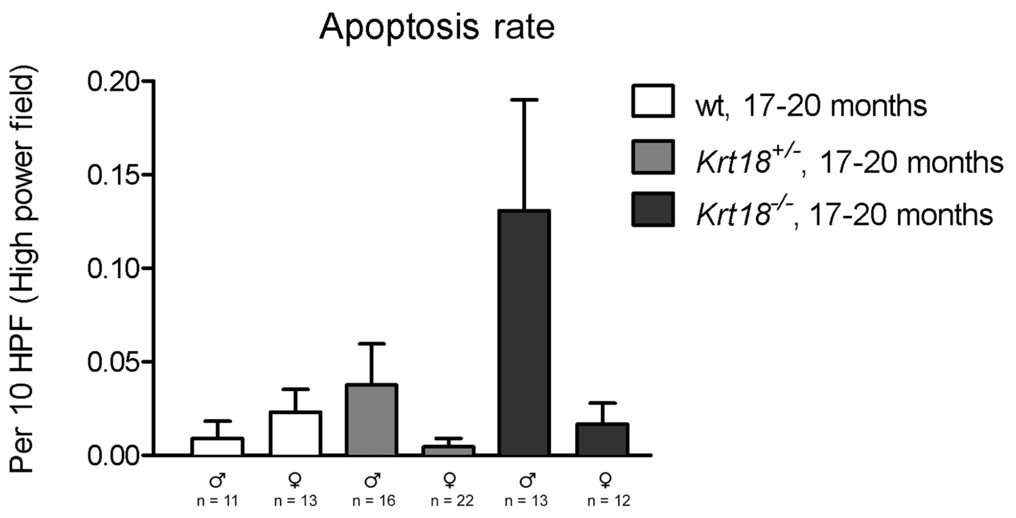

Supplementary Figure S1: Apoptotic rates of non-neoplastic livers of 17-20-months-old wt, *Krt18*$^{+/-}$ and *Krt18*$^{-/-}$ mice. Apoptotic bodies are rare in livers of aged wt and *Krt18*$^{+/-}$ male and female mice, but slightly more frequent in livers of male *Krt18*$^{-/-}$ mice.

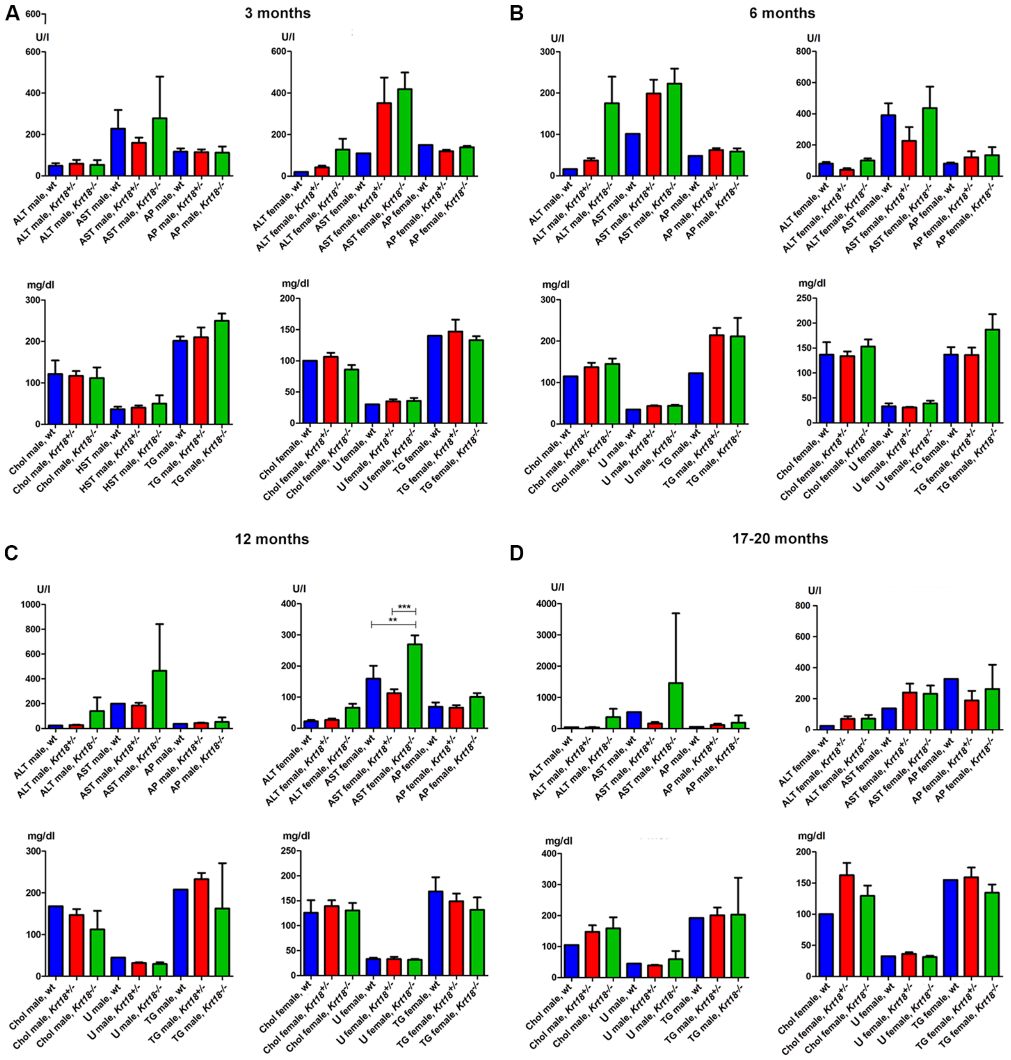

Supplementary Figure S2: Serum parameters show no significant differences between aged wt, *Krt18*$^{+/-}$ and *Krt18*$^{-/-}$ mice. Determination of blood serum from 3-, 6-, 12- and 17-20-months-old male and female mice. All values do not show statistically significant changes. Data are presented in U/l or mg/dl blood serum, results are shown as mean, error bars indicate SD or SEM *, **, *** indicate statistical significance (two way ANOVA with Bonferroni post-test): *= $p < 0.05$; **= $p < 0.001$; ***= $p < 0.0001$.

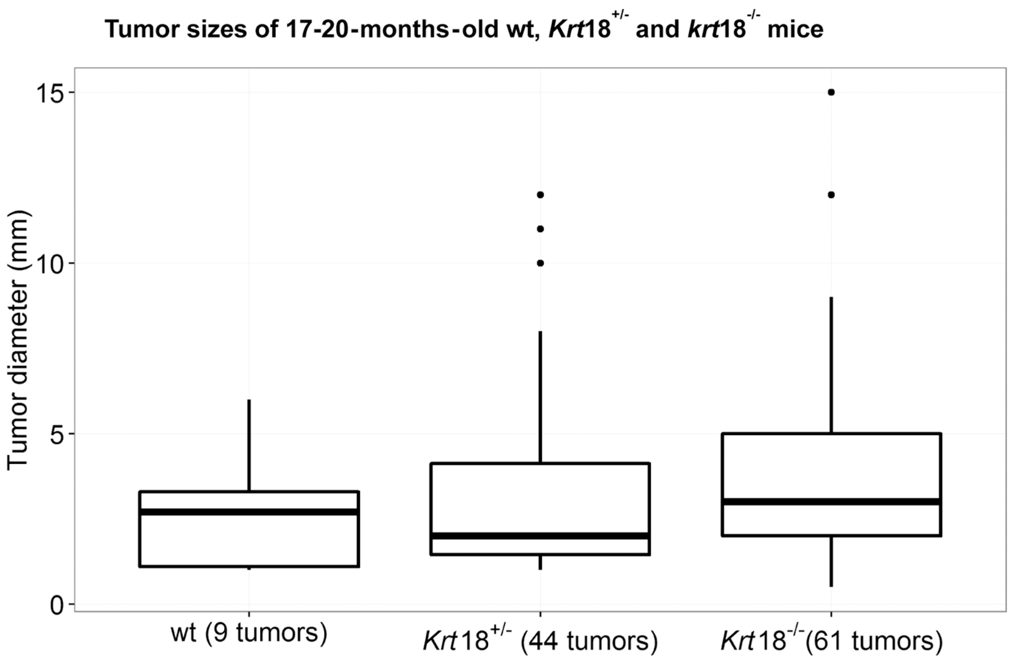

Supplementary Figure S3: Tumor sizes of 17-20-months-old wt, *Krt18*$^{+/–}$ and *Krt18*$^{–/–}$ mice. Accumulative Box-plots of tumor diameters in each genotype group.

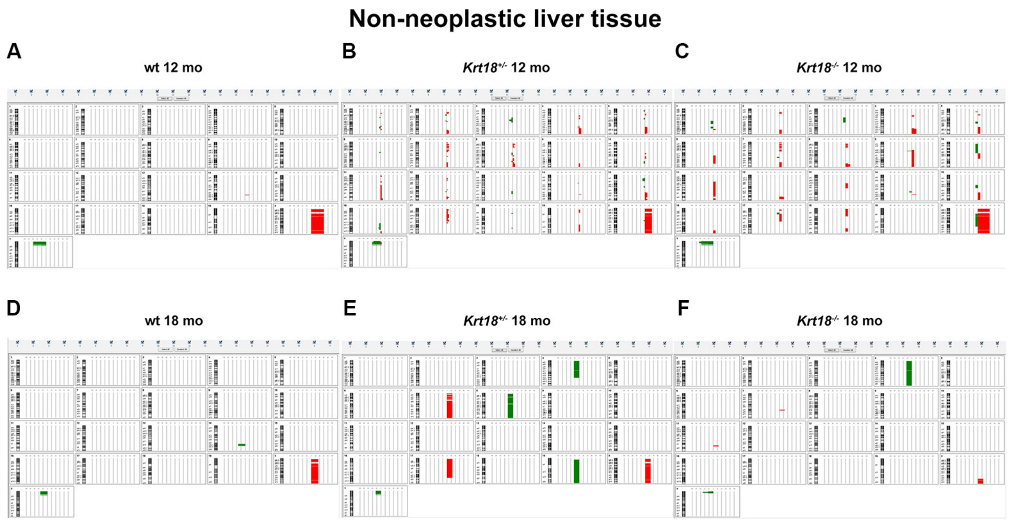

Supplementary Figure S4: Chromosomal aberration profile of non-neoplastic livers of 12- and 17-20-months-old wt, *Krt18*$^{+/–}$ and *Krt18*$^{–/–}$ mice. Accumulative penetrance plot of chromosomal imbalances in wt, *Krt18*$^{+/–}$ and *Krt18*$^{–/–}$ mice. Each sub-graph shows the q-arm of one chromosome specified by a number. The horizontal axis in each sub-graph indicates the number of samples, while the vertical axis shows the position on the q-arm for each chromosome. Chromosomal amplifications are indicated in red on the right part of each sub-graph and deletions are indicated in green on the left part of each sub-graph. (**A**–**F**) aCGH analyses reveal very few chromosomal aberrations in wt livers while *Krt18*$^{+/–}$ and *Krt18*$^{–/–}$ livers without any morphologic signs of tumor formation reveal frequent chromosomal aberrations. 12-months-old mice: livers of 6 wt (3 males, 3 females) mice; 18 *Krt18*$^{+/–}$ (11 males, 7 females) mice; 9 *Krt18*$^{–/–}$ (4 males, 5 females) mice. 17-20-months-old mice: livers of 3 wt (2 males, 1 female) mice; 4 *Krt18*$^{+/–}$ (4 males, 0 female) mice; 4 *Krt18*$^{–/–}$ (4 males, 0 female) mice.

Supplementary Table S1: Serum parameters without any significant differences between aged wt, *Krt18*$^{+/-}$ and *Krt18*$^{-/-}$ mice

3-months

male	ALT	AST	AP	Chol	U	TG
wt	3	3	3	3	3	3
Krt18$^{+/-}$	9	9	9	9	9	9
Krt18$^{-/-}$	3	3	3	3	3	3

female	ALT	AST	AP	Chol	U	TG
wt	1	1	1	1	1	1
Krt18$^{+/-}$	7	7	7	7	7	7
Krt18$^{-/-}$	6	6	6	6	6	6

6-months

male	ALT	AST	AP	Chol	U	TG
wt	5	5	5	5	5	5
Krt18$^{+/-}$	17	17	17	17	12	17
Krt18$^{-/-}$	6	6	6	6	5	6

female	ALT	AST	AP	Chol	U	TG
wt	3	3	3	3	3	3
Krt18$^{+/-}$	5	5	5	5	5	5
Krt18$^{-/-}$	5	5	5	5	5	5

12-months

male	ALT	AST	AP	Chol	U	TG
wt	2	2	2	2	1	2
Krt18$^{+/-}$	10	10	10	10	9	10
Krt18$^{-/-}$	2	2	2	3	2	2

female	ALT	AST	AP	Chol	U	TG
wt	5	5	5	5	4	5
Krt18$^{+/-}$	11	11	11	11	9	11
Krt18$^{-/-}$	5	5	5	5	4	5

17-20-months

male	ALT	AST	AP	Chol	U	TG
wt	2	2	2	2	2	2
Krt18$^{+/-}$	10	10	10	10	9	10
Krt18$^{-/-}$	3	3	3	3	3	3

female	ALT	AST	AP	Chol	U	TG
wt	2	2	2	2	2	2
Krt18$^{+/-}$	11	11	11	11	11	11
Krt18$^{-/-}$	10	10	10	10	10	10

Supplementary Table S2: Multiplicity of tumors in mice

A

	**Number of tumors**	**Number of mice**
**wt**	**9**	**7**
**males**	4	3
**females**	5	4
Krt18 $^{+/-}$	**44**	**21**
**males**	36	16
**females**	8	5
**Krt18** $^{-/-}$	**66**	**21**
**males**	44	12
**females**	22	9

B

	Krt18 $^{+/-}$	***Krt18*** $^{-/-}$
**wt**	0.23	0.04
Krt18 $^{+/-}$	–	0.85

C

	**wt females**	***Krt18*** $^{+/-}$ **males**	***Krt18*** $^{+/-}$ **females**	***Krt18*** $^{-/-}$ **males**	***Krt18*** $^{-/-}$ **females**
**wt males**	>.99	.14	.65	.03	.32
**wt females**	-	.18	.71	.03	.36
Krt18 $^{+/-}$ **males**	-	-	.27	>.99	.55
Krt18 $^{+/-}$ **females**	-	-	-	.07	.58
Krt18 $^{-/-}$ **males**	-	-	-	-	.34

Supplementary Table S3: Tumor sizes in mice

	Krt18 $^{+/-}$	***Krt18*** $^{-/-}$
**wt**	.66	.27
Krt18 $^{+/-}$	-	.12

Supplementary Table S4: Number of mice with non-tumor regions investigated for chromosomal aberrations by aCGH analysis

Number of mice with non-tumor regions investigated for chromosomal aberrations		
**Age**	**Genotype**	**Number of mice with chromosomal aberrations**
12-months	wt	0/6 (3 males, 3 females)
	Krt18$^{+/-}$	2/18 (11 males, 7 females)
	Krt18$^{-/-}$	2/9 (4 males, 5 females)
17–20 months	wt	0/3 (2 males, 1 female)
	Krt18$^{+/-}$	1/4 (4 males, 0 female)
	Krt18$^{-/-}$	1/4 (4 males, 0 female)

Supplementary Table S5: Basic immunohistochemistry protocol description

Antigen of antibody	Species	Dilution of primary antibody	Secondary antibody	Secondary Reagent	Dilution of secondary antibody	Company primary antibody	Company secondary antibody	Antigen retrieval
Sqstm1/p62	guinea pig	1:200	Rab-a-gp Igs	HR peroxidase conjugated undiluted reagent ready to use	1:100	Progen, Heidelberg, Germany (#GP62-C)	Dako	MW, pH 6.0
GS	mouse	1:5000				Millipore Temecula, Temecula, CA, USA (#AB1783)	Ventana	CC1
Ki67p	rabbit	1:1000		HR peroxidase conjugated undiluted reagent ready to use		Novocastra, Newcastle, UK (#PA0230)	Dako	MW, pH 6.0
K19	rabbit	1:500		HR peroxidase conjugated undiluted reagent ready to use		Epitomics, Burlingame, CA, USA (#3863-1)	Dako	WB, pH 6.0
Ubiquitin	rabbit	1:300		HR peroxidase conjugated undiluted reagent ready to use		Dako, Glostrup, Denmark (#ZO45801)	Dako	P
K8(Troma-3)	rat	1:50	Rab-a-rat Igs	HR peroxidase conjugated undiluted reagent ready to use	1:100	Self genarated in Hybridoma cells (Investigator:Brulet, P./kemler, R.)	Dako	MW, pH 6.0
Beta-catenin	mouse	1:100		SA		Becton Dickinson, Franklin Lakes, USA (#610153)	Empire Genomics	A

Immunohistochemistry for GS and beta-catenin was performed using a Ventana Immunostainer XT with ultra view DAB detection kit with cell conditioning solution (CC1,Ventana) for epitope retrieval. Rabbit (rab)-a-Ki67, rab-a-CK19, rab-a-Ubiquitin, guinea pig (gp)-a-p62CT and rat-a-CK8 were performed on the Dako immunostainer classic with the detection kit Dako K5007 (DAKO-REAL™ En-Vision™) using Dako 3-amino-9-ethylcarbazole (AEC) substrate (ready to use) as chromogen. Respective antibodies and their dilutions are used as in the table. The immunohistochemistry for beta –catenin was performed by using a standard protocol. P: pronase treatment, MW: Microwave, WB: water bath, A : autoclave, SA for beta-catenin: part of IDSTM003. Labeled polymer (LP), DAKO-REAL™ En-Vision™ Detection system, Peroxiadse/ DAB+, Rabbit/Mouse used for detection except for GS and beta-catenin.
